# Randomized clinical trial and meta-analysis of the impact of a fibrin sealant patch on pancreatic fistula after distal pancreatectomy: CPR trial

**DOI:** 10.1093/bjsopen/zrab001

**Published:** 2021-06-12

**Authors:** T H Mungroop, N van der Heijde, O R Busch, I H de Hingh, J J Scheepers, M G Dijkgraaf, B Groot Koerkamp, M G Besselink, C H van Eijck

**Affiliations:** Department of Surgery, Cancer Centre Amsterdam, Amsterdam UMC, University of Amsterdam, Amsterdam, the Netherlands; Department of Surgery, Cancer Centre Amsterdam, Amsterdam UMC, University of Amsterdam, Amsterdam, the Netherlands; Department of Surgery, Cancer Centre Amsterdam, Amsterdam UMC, University of Amsterdam, Amsterdam, the Netherlands; Department of Surgery, Catharina Hospital, Eindhoven, the Netherlands; Department of Surgery, Erasmus MC, Rotterdam, the Netherlands; Department of Clinical Epidemiology, Biostatistics and Bioinformatics, Amsterdam UMC, University of Amsterdam, Amsterdam, the Netherlands; Department of Surgery, Erasmus MC, Rotterdam, the Netherlands; Department of Surgery, Cancer Centre Amsterdam, Amsterdam UMC, University of Amsterdam, Amsterdam, the Netherlands; Department of Surgery, Erasmus MC, Rotterdam, the Netherlands

## Abstract

**Background:**

Postoperative pancreatic fistula (POPF) remains the main cause of morbidity in patients after distal pancreatectomy. The objective of this study was to investigate whether an absorbable fibrin sealant patch could prevent POPF after distal pancreatectomy.

**Methods:**

A multicentre, patient-blinded, parallel-group randomized superiority trial was performed in seven Dutch hospitals. Allocation was done using a computer-generated randomization list with a 1 : 1 allocation ratio and concealed varying permuted block sizes. Pancreatic stump closure with a fibrin patch was compared with standard treatment in patients undergoing distal pancreatectomy. The primary endpoint was the development of grade B/C POPF. A systematic review and meta-analysis was performed which combined the present findings with all available evidence.

**Results:**

Between October 2010 and August 2017, 247 patients were enrolled. Fifty-four patients (22.2 per cent) developed a POPF, 25 of 125 patients in the patch group *versus* 29 of 122 in the control group (20.0 *versus* 23.8 per cent; *P *=* *0·539). No related adverse effects were observed. In the meta-analysis, no significant difference was seen between the patch and control groups (19.7 *versus* 22.0 per cent; odds ratio 0.89, 95 per cent c.i. 0.60 to 1.32; *P *=* *0·556).

**Conclusion:**

Application of a fibrin patch to the pancreatic stump does not reduce the incidence of POPF in distal pancreatectomy. Future studies should focus on alternative fistula mitigation strategies, considering pancreatic neck thickness and duct size as risk factors. Trial registration number NL5876 (Netherlands Trial Registry).

## Graphical Abstract

Postoperative pancreatic fistula (POPF) remains a major concern after distal pancreatectomy. Although stump closure with a fibrin sealant patch has shown promising results, patch application did not reduce the incidence of POPF in this multicentre, patient-blinded RCT.

## Introduction

Postoperative pancreatic fistula (POPF) remains the main cause of morbidity after distal pancreatectomy (DP). No clear guidelines exist for closure of the pancreatic stump, or how to prevent POPF after DP. The use of absorbable fibrin patches has been investigated in pancreatic surgery for several years^1^. At the time of the start of this study, available reports suggested a possible benefit of fibrin patches in terms of reduction in POPF^2^^,^^3^. In a non-blinded multicentre RCT^4^, the observed risk reduction of 6 (95 per cent c.i. –14 to +1.4) per cent cannot completely rule out a clinically relevant effect of fibrin patches in reducing POPF.

In 2016, the International Study Group of Pancreatic Surgery (ISGPS)^5^ updated its classification of POPF. The clinically irrelevant grade A POPF was redefined as biochemical leak, and the definition of grade B and C fistula was optimized to be more objective and specific. Stump closure with a fibrin patch has not been investigated using the updated ISGPS 2016 classification. Therefore, the aim of this multicentre and patient-blinded RCT was to investigate whether an absorbable fibrin sealant patch could prevent significant POPF after DP.

## Methods

The CPR (closure of the pancreatic remnant after distal pancreatectomy) trial was designed as a multicentre, investigator-initiated, patient-blinded, parallel-group, randomized superiority trial. The study was conducted in seven Dutch hospitals belonging to the Dutch Pancreatic Cancer Group and followed the CONSORT guidelines^6^. The CPR trial was investigator-driven and done in accordance with the principles of the declaration of Helsinki^7^. It was approved by the Medical Ethical Committee (number MEC13-433) of Erasmus MC (Rotterdam, the Netherlands), and registered in the Netherlands Trial Registry (NL5876). A data monitoring safety board was not set up, because safety risks were limited. The study protocol with amendments is available in *Appendices 1* and *2*.

Adult patients undergoing open or minimally invasive DP were eligible for inclusion, if they had an expected survival time of at least 12 months. Exclusion criteria were: current immunosuppressive therapy, recent chemotherapy (less than 2 weeks before surgery), psychiatric/neurological disease, and/or drug or alcohol abuse. All patients gave written informed consent before surgery.

### Randomization, treatment allocation, and blinding

A central study coordinator allocated patients randomly during surgery using a concealed randomization list. This study coordinator was involved only in group assignment of trial subjects. The randomization list was computer-generated with a 1 : 1 allocation ratio, and concealed varying permuted block sizes of two, four, six, or eight patients. No stratification was applied. Randomization was done during surgery after the pancreas had been transected. Patients remained blinded to the group allocation for at least 12 months after surgery.

### Intervention

In both groups, the pancreas was transected using a stapler or surgical scalpel with suturing. In the fibrin patch group, a fibrin and thrombin-coated collagen patch (TachoSil^®^; Takeda Pharmaceutical Company, Tokio, Japan) was placed to cover the transection surface, including an overlap on the pancreas. All participating surgeons had received a video demonstrating this procedure. In the control group, patients received standard treatment without a fibrin patch. In both groups, no other additional stump closure techniques were allowed. One or more intra-abdominal surgical drains were placed near the pancreatic remnant. On the third postoperative day, amylase levels were measured in serum and drain fluid.

### Outcomes

The initial primary endpoint was the development of pancreatic fistula, defined according to the ISGPS classification^8^, in the first 90 days after operation. During the inclusion period of the trial, an updated ISGPS classification for POPF was published^5^. This new definition and grading system became the new standard from 2017 onwards. An external validation study concluded that the updated POPF definition showed improved discrimination between grades and should therefore be used to report POPF after DP^9^. This led to the decision to change the primary outcome to development of POPF (grade B or C) according to the ISGPS 2016 classification^5^. This was done before data collection had been completed and analysis had commenced. A drain amylase concentration of more than three times the upper level of the institutional normal value was used to define a biochemical leak. Grade B POPF is defined by a biochemical leak with persistent drainage (more than 21 days), a clinically relevant change in management, percutaneous or endoscopic drainage, angiographic procedure for bleeding, or signs of infection without organ failure. Grade C POPF is a fistula leading to reoperation, organ failure, or death. Secondary endpoints included POPF (more than 3-fold increase in drain amylase level on or after day 10), graded accorded to the Strasberg definition (grades 1–5)^10^. Other secondary endpoints included: duration of operation, blood loss, need for intraoperative transfusion, time to return to normal diet, postoperative complications (delayed gastric emptying (DGE), postpancreatectomy haemorrhage (PPH), pneumonia, other pulmonary complications, myocardial infarction, other cardiac complications, deep venous thrombosis, stroke, urinary tract infection, intra-abdominal abscess, sepsis, wound infection/dehiscence, peritonitis), reinterventions, duration of hospital stay, reoperation, readmission, and mortality (in hospital and within 90 days). DGE and PPH were scored according to the appropriate ISGPS definitions^11^^,^^12^. Grade B or C DGE or PPH was considered clinically relevant. Pancreatic thickness and pancreatic duct size were measured on preoperative imaging (mostly CT) at the pancreatic neck. This was done at the level of the confluence of the portosplenomesenteric veins, in the anterior–posterior plane, so not necessarily perpendicular to the pancreatic surface. All endpoints were assessed up to 90 days after surgery.

### Statistical analysis

The sample size was calculated based on data from 112 consecutive patients who had DP treated in Erasmus MC from 2006 to 2009. In this cohort, grade B/C POPF occurred in 30 per cent of patients, similar to the rate reported in a meta-analysis^13^ published before the start of the present study. An absolute reduction of 15 per cent (relative risk reduction 50 per cent) in the intervention group was chosen, which was also a pragmatic choice to obtain a realistic sample size. Using a power of 80 per cent (1 – β) and a two-sided α level of 0.05, 118 patients were needed in each arm. Assuming a dropout of 6 per cent, the total sample size needed was calculated to be 250 patients.

Analyses were done according to the intention-to-treat principle. The normality of distribution was checked by visual inspection of histograms. For continuous variables, normally distributed data were summarized as mean(s.d.), and non-normally distributed data as median (i.q.r.), with testing for differences between groups using Student’s *t* test and Mann–Whitney *U* test respectively. Dichotomous data are presented with percentages, and Fisher’s exact test was used for all analysis of proportions. *P* < 0·050 was considered statistically significant. The logistic regression analysis, analysis of surgical approach (minimally invasive *versus* open), method of stump closure, and the meta-analysis were exploratory analyses. All other analyses were confirmatory. A logistic regression model was used to assess the effect of the fibrin patch in the presence of known risk factors: pancreatic thickness, pancreatic duct size, pathology, and method of stump closure. The goal of this analysis was both to explore whether known risk factors were predictive in the present data set, and to reduce confounding bias in analysis of the primary endpoint. The selection of these risk factors was based on currently available literature^14–17^. Because some studies reported possibly higher rates of POPF in minimally invasive compared with open DP^18^, an ad hoc logistic regression analysis was done to test the interaction of the fibrin patch and surgical approach. Statistical analysis was undertaken using SPSS^®^ version 22.0 (IBM, Armonk, New York, USA).

### Systematic review and meta-analysis

During the study interval, other RCTs assessed the impact of fibrin patches (TachoSil^®^) after DP. A meta-analysis of all available RCTs of fibrin patches in DP was done, according to the PRISMA guidelines^19^, to pool these data with those from the present study.

#### Search

A systematic literature search was conducted in Embase, Cochrane Central Register of Controlled Trials, and PubMed databases to search for RCTs on this topic up to April 2020. Search terms were based on procedure (pancreatectomy) and intervention (TachoSil^®^). The search in PubMed was as follows: ‘(sealant OR sealing OR TachoSil OR TachoComb OR patch) AND ‘distal pancreatect*’. Similar search strategies were used for the Cochrane Library and Embase. The major clinical trial registries (ClinicalTrials.gov: http://clinicaltrials.gov/; International Clinical Trials Registry Platform Search Portal: http://apps.who.int/trialsearch/) were checked for any ongoing trials. The term ‘randomized trial’ was not included in the search strategy to ensure that no clinical trials were missed during the screening process. Titles, abstracts, and full-text articles were all independently assessed by two authors to establish eligibility. References of included studies were screened manually for possible additional studies.

#### Risk of bias

The Cochrane risk-of-bias tool^20^ was used to assess risk of bias in the individual studies by two reviewers independently. As fewer than 10 studies were included in this analysis, it was not possible to assess publication bias with funnel plots^21^.

#### Meta-analysis

Data analyses were performed using Comprehensive Meta-Analysis (Biostat (C), Englewood (NJ), United States), version 3.0 (CMA 3.0) software. Categorical data are presented as frequencies and percentages. A DerSimonian and Laird random-effects model was used to pool the data^22^. The numbers of grade B/C POPFs and sample size per group were used to calculate odds ratios (ORs). The incidence of POPF in each study was used as originally reported (2005 or 2016 ISGPS definition). The *I*^2^ statistic was used to assess between-study heterogeneity. An *I*^2^ value of 0–40 per cent was interpreted as low, 30–60 per cent as moderate, 50–90 per cent as high, and 75–100 per cent as considerable heterogeneity^23^. A forest plot was used to visualize the data. Statistical significance was set at *P *<* *0·050.

## Results

**Fig. 1:**
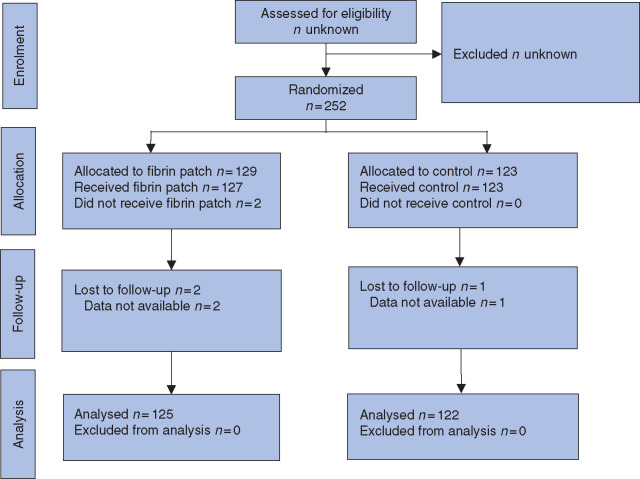
CONSORT diagram for the trial

Between 11 October 2010 and 7 August 2017, 252 patients were randomized. Randomization was performed too early for two patients, and DP was ultimately not carried out because metastases were detected. Three patients were lost to follow-up (no data available), and 247 patients were included in the final analysis (*Fig. 1*). The fibrin patch group consisted of 125 patients and there were 122 patients in the control group. Baseline characteristics, intraoperative variables, and pathological outcomes were comparable between groups (*Tables 1* and *2*). More than half of the patients were women (56 per cent) and the median age was 62 years. Minimally invasive DP was performed in 37 per cent of patients. Somatostatin analogues (such as octreotide) were used in 16 per cent of patients: in 15 (12 per cent) in the patch group *versus* 24 (20 per cent) in the control group (*P *= 0.117). The most common indications for DP were adenocarcinoma (63 patients, 26 per cent) and neuroendocrine tumours (58 patients, 23 per cent). Median duration of operation was 258 (i.q.r. 203–333) min.

**Table 1:** Patient characteristics

	**Fibrin patch** **(*n* = 125)**	**Control** **(*n* = 122)**
Age (years)*	62 (48–69)	63 (53–69)
**Sex ratio (M : F)**	57 : 68	52 : 70
BMI (kg/m^2^)*	26 (22–28)	25 (22–28)
Pancreatic neck thickness (mm)*	13 (11–16)	13 (10–16)
Pancreatic duct size (mm)*	2 (2–3)	2 (1–3)
Karnofsky score*	90 (80–100)	90 (80–90)
**History of pancreatic or biliary surgery**	23 (18)	27 (22)
**Co-morbidity**		
Cardiovascular	27 (22)	30 (25)
Hypertension	34 (27)	28 (23)
Stroke	8 (6·4)	6 (4·9)
Diabetes	15 (12)	22 (18)
Pulmonary	18 (14)	29 (24)
**Recent diabetes^†^**	6 (4·8)	3 (2·5)

Values in parentheses are percentages unless indicated otherwise;

*values are median (i.q.r.).

^†^Development of diabetes in the 12 months before surgery.

**Table 2:** Intraoperative and pathological outcomes

	**Fibrin patch** **(*n* = 125)**	**Control** **(*n* = 122)**
Duration of operation (min)*	256 (199-336)	261 (208-332)
**Minimally invasive approach**	49 (39)	43 (35)
**Splenectomy**	68 (54)	72 (59)
**Type of transection**		
Stapler	93 (80)	82 (72)
	93 (74)	82 (67)
Hand-sewn	24 (20)	32 (28)
	24 (19)	32 (26)
	8 (6)	8 (7)
**Pathology**		
Solid neoplasm		
Pancreatic adenocarcinoma	24 (19)	39 (32)
Neuroendocrine tumour	28 (22)	30 (25)
Other solid neoplasm	8 (6·4)	3 (2·5)
Cystic lesion		
IPMN	23 (18)	18 (15)
Other cystic lesion	13 (10)	11 (9)
Other	29 (23)	21 (17)

Values in parentheses are percentages unless indicated otherwise;

*values are median (i.q.r.). IPMN, intraductal papillary mucinous neoplasm.

### Outcomes

#### Primary endpoint

Overall, the rate of grade B/C POPF was 22 per cent: 25 of 125 patients (20 per cent) in the fibrin patch group *versus* 29 of 122 (24 per cent) in the control group (*P *=* *0.539) (*Table 3*). In the logistic regression model, application of a fibrin patch was not a statistically significant predictor of grade B/C POPF (*P *=* *0**·**473) (*Table S1*) in the presence of pancreatic neck thickness, pancreatic duct size, and pathology (malignant *versus* non-malignant). Independent risk factors for POPF were increasing pancreatic neck thickness (OR 1**·**19 (95 per cent c.i. 1**·**10 to 1**·**30) per mm increase) and increasing pancreatic duct size (OR 1·68 (1·22 to 2**·**32) per mm increase) (*Table S1*). The original primary endpoint was analysed and reported in *Table S2.*

**Table 3:** Primary and secondary endpoints

	**Fibrin patch** **(*n *=* *125)**	**Control** **(*n *=* *122)**	**Mean difference** ^†,§^	***P*** ^¶^
**Postoperative pancreatic fistula**	25 (20)	29 (24)	4 (–7, 14)	0·539
Grade B	23 (18)	25 (20)		
Grade C	2 (1.6)	4 (3·3)		
Time to drain removal (days)*	3 (4–7)	5 (3–10)	2 (–1, 7)	0·336^#^
**Major morbidity (Clavien–Dindo grade ≥ III)**	30 (24)	36 (30)	6 (–6, 17)	0·389
**Delayed gastric emptying**	4 (3·2)	7 (5·7)	3 (–1, 8)	0·372
**Postpancreatectomy haemorrhage**	2 (1·6)	6 (4·9)	3 (–1, 8)	0·170
Intraoperative blood loss (ml)*	300 (123–800)	565 (150–1300)	134 (–156, 426)	0·168^#^
**Reoperation**	4 (3·2)	12 (9·8)	7 (0, 13)	0·040
Bowel perforation	3 (2·4)	5 (4·1)		0·496
Haemorrhage	0 (0)	3 (2·5)		0·119
Other reasons^‡^	1 (0·8)	4 (3·3)		
Duration of hospital stay (days)*	7 (5–9)	8 (6–11)	2 (0, 4)	0·025^#^
**Readmission**	23 (18)	30 (25)	6 (–4, 16)	0·279
**In-hospital mortality**	2 (1·6)	5 (4·1)	3 (–2, 7)	0·277
**90-day mortality**	2 (1·6)	6 (4·9)	3 (–1, 8)	0·168

Values in parentheses are percentages unless indicated otherwise;

*values are median (i.q.r.) and

^†^values in parentheses are 95 per cent confidence intervals.

^‡^Fibrin patch group: removal of broken abdominal drain (1); control group: fascial dehiscence (2), adhesiolysis (1), persistent pain and paralytic ileus, but no abnormalities during reoperation (1).

^§^For all continuous variables, normality was assumed for this analysis, even for those with a non-normal distribution.

^¶^Fisher’s exact test, except #Mann–Whitney *U* test.

#### Secondary endpoints

The incidence of major morbidity (Clavien–Dindo grade at least III) did not differ between groups (*Table 3*). No difference was found regarding DGE and PPH (*Table 3*), or in other complications (*Table S3*). Hospital stay was shorter in the fibrin patch group. Reoperations were performed in four patients (3.2 per cent) in the fibrin patch group *versus* 12 (9.8 per cent) in the control group (*P *=* *0·040), and 23 (18 per cent) *versus* 30 (25 per cent) respectively were readmitted to hospital (*P *=* *0·279). The 90-day mortality rate did not differ significantly between groups: 2 of 125 (1.6 per cent) *versus* 6 of 122 (4·9 per cent) (*P *=* *0·168). Deaths of three patients were related to POPF: 0 (0 per cent) *versus* 3 (2·5 per cent) in patch *versus* control group. There was no difference between groups regarding day of drain removal. No association was found between surgical technique and fibrin patch regarding the development of POPF (*P *=* *0·666, ad hoc logistic regression). Ad hoc logistic regression also showed that method of stump closure was not a significant predictor of POPF (*P *=* *0·504).

#### Systematic review and meta-analysis

**Fig. 2:**
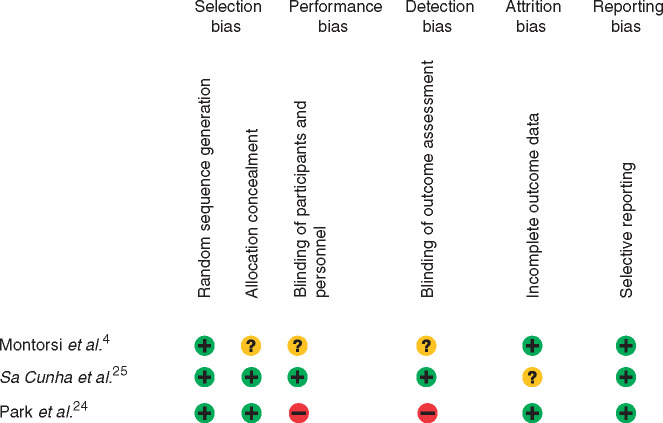
Risk-of-bias analysis for studies included in systematic review +, Low risk of bias; –, high risk of bias; ?, unclear risk of bias.

**Fig. 3:**
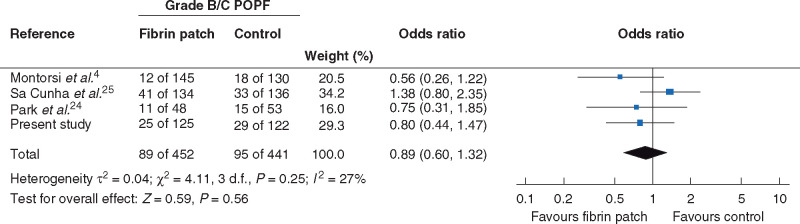
Meta-analysis of impact of fibrin patch on rate of gradeB/C postoperative pancreatic fistula A Mantel–Haenszel random-effects model was used for meta-analysis. Odds ratios are shown with 95 per cent confidence intervals. POFP, postoperative pancreatic fistula.

A total of 241 studies were identified, which were screened based on title and abstract, after which 234 studies were excluded as they did not meet the inclusion criteria. Of seven studies that remained, four were excluded after full-text assessment. Two studies were excluded owing to the design (not an RCT), and the other two used fibrin glue instead of a fibrin patch. As the comparability of a fibrin patch and fibrin glue is uncertain, only fibrin patch studies were included. In total, three studies^4^^,^^24^^,^^25^ comparing TachoSil^®^ with a control group were included in a meta-analysis *(Fig. S1*), along with data from the present trial. The risk of bias of included studies is summarized in *Fig. 2*. This risk was regarded as moderate in all of the included trials. All trials used the definition developed by the ISGPS. In two studies^4^^,^^24^, the primary outcome was the occurrence of POPF, whereas the other two had clinically relevant POPF as primary outcome. Only the present study used the new ISPGF classification. Development of clinically relevant POPF was analysed in the meta-analysis. There was no statistically significant difference between the fibrin patch and control groups in the rate of grade B/C POPF: 89 of 452 (19.7 per cent) *versus* 95 of 441 (22 per cent) respectively (OR 0·89, 95 per cent c.i. 0·60 to 1·32; *P *=* *0·556) (*Fig. 3*). There was low heterogeneity between studies (*I*^2^ = 27 per cent).

## Discussion

In this multicentre patient-blinded RCT, grade B/C POPF developed in 22 per cent of patients after DP in experienced centres. No significant reduction in POPF was seen with application of a fibrin patch. After adjusting for known risk factors, no beneficial effect of the fibrin patch was noted. The reoperation rate was lower in the fibrin patch group, although specific indications for reoperation did not differ much between groups. The systematic review and meta-analysis, which combined the present results with those from other available RCTs on this subject, confirmed that fibrin patches do not decrease the incidence of POPF in DP.

The overall 22 per cent rate of POPF after DP signifies the relevance of this complication and justifies the rationale of this study. Despite numerous studies on this topic, there is no consensus regarding the optimal method of stump management in DP. Three randomized trials^4^^,^^24^^,^^25^ have addressed the role of an absorbable fibrin patch in DP. In two^4^^,^^24^ of these, patients were not blinded to the treatment allocation. Patients’ knowledge of the study group assignment does not directly influence objective measures, such as drain amylase values. However, patient expectations related to the group assignment may influence recovery parameters and self-reporting of symptoms. Thus, blinding should be done in RCTs, if feasible, to reduce measurement bias. All previous trials used the ISGPS 2005 classification of POPF^8^, whereas the present multicentre RCT used the updated ISGPS 2016 definition. In one of the previous studies^25^, a total of 45 centres participated to include 270 patients, with the potential for wide heterogeneity in clinical standards between centres.

Although POPF rates were similar in the two groups in the present study, a shorter hospital stay and lower reintervention rate were observed in the fibrin patch group. The shorter hospital stay could be explained by the lower reoperation rate. As patch use was not a protective factor for POPF in the multivariable analysis, this seems coincidental.

Increasing pancreatic thickness and pancreatic duct size were independent prognostic factors for POPF. The relationship between duct size and POPF risk is contrary to that for pancreatoduodenectomy, where larger duct size reduces the risk of POPF^26^. Further research is needed to further validate this predictor, as measurements on preoperative imaging might be difficult to replicate exactly. Increasing pancreatic thickness has been identified as a risk factor for POPF in DP before^16^, and can be explained by greater difficulty in closing the pancreatic stump. This can cause crushing of the pancreatic cut surface, especially when a stapler device is used. The findings can aid in performance of risk-adjusted analyses in studies of DP^27^.

Numerous fistula mitigation strategies in DP have been proposed, such as fibrin patch application, no drain *versus* routine drainage, and hand-sewn *versus* stapler stump closure^28^^,^^29^. Only pasireotide was successful in a large single-centre randomized trial^30^ published in 2014, both in DP as well as pancreatoduodenectomy. A more recent RCT^31^ compared pasireotide with hydrocortisone in patients at high risk of pancreatic fistula; in assessment of this effect in patients undergoing DP, the POPF rate was lower in the pasireotide group (37 *versus* 67 per cent; *P* = 0.02). However, the old definition of POPF was used; when only grade B/C POPF was assessed, there was no significant difference (13 *versus* 20 per cent; *P* = 0.488) . Follow-up studies have questioned the value of this drug in DP^32^.

The present study has some limitations. First, more patients had non-malignant lesions in the fibrin patch group (19 *versus* 32 per cent), which may have biased the results. However, lack of benefit of a fibrin patch was confirmed in multivariable analysis that adjusted for malignant *versus* non-malignant lesions. Second, this study was conducted over a relatively long interval (2010–2017), during which minimally invasive DP was implemented in the Netherlands, as well as enhanced recovery pathways. Additionally, a new definition and grading system for POPF was published, which led to an adjustment in the primary outcome. Although a shorter period of inclusion would have been preferred, DP is performed less commonly than pancreatoduodenectomy, and fewer patients were available for the study than expected. Because of the randomized design, the authors feel this did not influence the study outcomes negatively. Finally, only the patients were blinded to the group allocation. The 90-day mortality rate (3 per cent) may seem slightly higher than that in more recent reports^18^, but can be explained by the study starting 2010. Future studies should focus on novel fistula mitigation strategies, especially in high-risk patients, because the absolute risk reduction can have the largest impact in this group. Promising novel strategies include perioperative hydrocortisone administration, which was successful in a randomized trial in DP^33^. Furthermore, botulinum toxin injection in the sphincter of Oddi showed a low rate of POPF in a non-randomized study^34^. Based on the results of this RCT and meta-analysis, POFP remains a relevant complication after DP and fibrin patches do not decrease the rate of POPF.

## Collaborators

The authors are grateful for the contributions made by the following collaborators: T. de Rooij (Amsterdam UMC, Amsterdam); M. Luyer (Catharina Hospital, Eindhoven); K. van Laarhoven (Radboud UMC, Nijmegen); M. Stommel (Radboud UMC, Nijmegen); R. de Kleine (UMC Groningen, Groningen); Q. Molenaar (UMC Utrecht, Utrecht).

## Supplementary Material

zrab001_Supplementary_Data
