# Supplementary material for: Randomized clinical trial and meta-analysis of the impact of a fibrin sealant patch on pancreatic fistula after distal pancreatectomy: CPR trial
Source: BJS Open. 2021 Jun 12;5(3):zrab001. doi: 10.1093/bjsopen/zrab001 (PMC8262074; doi:10.1093/bjsopen/zrab001)
Supplement: zrab001_Supplementary_Data [file zrab001_supplementary_data.zip › Appendixes.docx]

**Appendix 1. Incidence of other complications**

|  | **Fibrin Patch**  **(n = 125)** | **Control**  **(n = 122)** | ***P*** |
| --- | --- | --- | --- |
| Pneumonia | 12 (9·6) | 13 (11) | 0·835 |
| Other pulmonary complications | 9 (7·2) | 6 (4·9) | 0·596 |
| Myocardial infarction | 2 (1·6) | 1 (0·8) | >0·999 |
| Other cardiac complications | 6 (4·8) | 5 (4·1) | >0·999 |
| Deep venous thrombosis | 0 | 0 | - |
| Cerebrovascular accident | 0 | 0 | - |
| Urinary tract infection | 2 (1·6) | 6 (4·9) | 0·168 |
| Intra-abdominal abscess | 24 (19) | 20 (16) | 0·620 |
| Sepsis | 10 (8) | 8 (6·6) | 0·808 |
| Wound infection or dehiscence | 6 (4·8) | 10 (8·2) | 0·311 |
| Peritonitis | 2 (1·6) | 3 (2·5) | 0·681 |

*Values in parentheses are percentages.*

**Appendix 2. Risk factors for pancreatic fistula after distal pancreatectomy**

|  | **Univariate analysis** | |  | **Multivariate analysis** | |
| --- | --- | --- | --- | --- | --- |
|  | **No POPF (n = 193)** | **POPF (n = 54)** | ***P*** | **Odds ratio (95% CI^)** | ***P*** |
| Fibrin patch | 93 (48) | 29 (54) | 0·473 |  |  |
| Sex (male) | 79 (41) | 30 (56) | 0·057 |  |  |
| Age (years)* | 63 (55 - 67) | 62 (50 - 70) | 0·457 |  |  |
| Body-Mass Index (kg/m^2^)* | 25 (22 - 28) | 26 (24 - 29) | 0·474 |  |  |
| Pancreatic neck thickness (mm)* | 12 (10 - 15) | 16 (13 - 19) | <0·001 | 1**·**19 (1**·**10 -1**·**30) | <0·001 |
| Pancreatic duct size (mm)* | 2 (1 - 3) | 3 (2 - 4) | <0·001 | 1**·**68 (1**·**22 - 2**·**32) | 0·007 |
| Karnofsky score* | 90 (80 - 100) | 90 (80 - 90) | 0·123 |  |  |
| Malignant pathology | 48 (25) | 15 (28) | 0·665 |  |  |
| History of pancreatic or biliary surgery | 37 (19) | 13 (24) | 0·429 |  |  |
| Comorbidities | - | - |  |  |  |
| - Cardiovascular | 43 (22) | 14 (26) | 0·574 |  |  |
| - Hypertension | 50 (26) | 12 (20) | 0·581 |  |  |
| - Cerebrovascular accident | 12 (6·2) | 2 (3·7) | 0·485 |  |  |
| - Diabetes | 27 (14) | 10 (19) | 0·411 |  |  |
| - Pulmonary | 37 (19) | 10 (19) | 0·912 |  |  |
| Recent diabetes^†^ | 4 (2·1) | 5 (9·3) | 0·023 |  |  |

*Values in parentheses are percentages unless indicated otherwise; * values are median (i.q.r.); † development of Diabetes in the 12 months before surgery; ^ confidence interval.*

**Appendix 3. Prisma flow chart**

**Appendix 4.** Pancreatic Fistula according to the Strasberg Classification

|  | **Fibrin Patch (n = 125)** | **Control (n = 122)** | ***P*** |
| --- | --- | --- | --- |
| Postoperative pancreatic fistula (Strasberg) | **-** | **-** | 0.74 |
| - Grade I | 13 (10%) | 17 (14%) | - |
| - Grade II | 1 (0.8%) | 1 (0.8%) | *-* |
| - Grade III | 19 (15%) | 16 (13%) | *-* |
| - Grade IV | 0 | 1 (0.8%) | *-* |
| - Grade V | 0 | 1 (0.8%) | *-* |
