# Supplementary material for: Randomized clinical trial and meta-analysis of the impact of a fibrin sealant patch on pancreatic fistula after distal pancreatectomy: CPR trial
Source: BJS Open. 2021 Jun 12;5(3):zrab001. doi: 10.1093/bjsopen/zrab001 (PMC8262074; doi:10.1093/bjsopen/zrab001)
Supplement: zrab001_Supplementary_Data [file zrab001_supplementary_data.zip › 01-11-20 Mungroop - CPR trial Protocol Modifications.docx]

**Changes to the CPR trial study protocol**

- Primary endpoint of POPF is now reported as grade B/C POPF according to the new ISGPS 2016 classification. This was done since this became the new standard for reporting of POPF in pancreatic surgery studies. We have included the results according to the original primary endpoint in appendix 4.

- Inclusion is broadened to both laparotomy and laparoscopic distal pancreatectomy since the latter approach became implemented nationwide during the study period.

- Medical costs as a secondary endpoint. This analysis has not been done yet, since no clinical benefit was shown with the use of fibrin patches.

- A meta-analysis was added post-hoc, as suggested during the review process.
