# Supplementary material for: Randomized clinical trial and meta-analysis of the impact of a fibrin sealant patch on pancreatic fistula after distal pancreatectomy: CPR trial
Source: BJS Open. 2021 Jun 12;5(3):zrab001. doi: 10.1093/bjsopen/zrab001 (PMC8262074; doi:10.1093/bjsopen/zrab001)
Supplement: zrab001_Supplementary_Data [file zrab001_supplementary_data.zip › Closure of the pancreatic remnant- PROTOCOL.doc]

**Study Protocol**

***C*LOSURE OF THE *P*ANCREATIC *R*EMNANT AFTER DISTAL PANCREATECTOMY**

A prospective randomized controlled trial.

Principle investigators and writing committee

J.J.G. Scheepers

O.R.C. Busch

C.H.J. van Eijck

**CPR- trial**

**SUMMARY**

**Rationale:** Inappropriate closure of the pancreatic remnant after distal pancreatectomy remains a common source of morbidity. Pancreatic fistula and leakage are the most common and clinically relevant complications, and they are thought to depend on surgical technique and skill A variety of procedures have been recommended to reduce the frequency of pancreatic fistula. Results of previously described techniques after pancreatico-duodenectomy suggest that outcome with regard to pancreatic fistula can be improved using fibrinogen/thrombin coated collagen patch (TachoSil®, Nycomed, UK Ltd.) in carrying out a pancreaticojejunostomy. The proposed trial will compare a standard method of hand or stapled closure of the pancreatic remnant with or without the use of a collagen patch (TachoSil). If the collagen patch is effective in reducing pancreatic fistula and overall morbidity, it has a potential to improve quality of life and reduces medical costs.

**Objective:** The objective is to investigate the effect of Tachosil sealing of the pancreatic remnant after distal pancreatectomy on the development of pancreatic fistula. We aim to recommend a technique for optimal closure of the pancreatic remnant, leading to a lower incidence of pancreatic fistula. This will decrease hospital stay and substantially reduce medical costs.

**Study design:** Randomized controlled trial. Patients and medical personnel, except surgeons, are blinded for treatment allocation.

**Study population:** Patients will undergo explorative laparotomy and subsequent distal pancreatectomy will be asked to participate in the study. If a resection is to be carried out, based on preoperative findings, participants are eligible for randomization.

**Duration:** 3 years.

**Intervention:** The intervention is the placement of a collagen patch on the sutured or stapled pancreatic remnant, with a sutured/stapled closed only as control.

**Main study parameters:** Primary outcome parameter is the incidence of pancreatic fistula according to the grading system for pancreatic occlusion failure. Secondary outcome parameters are mortality, postoperative complications, length of hospital stay and medical costs.

**BACKGROUND AND RATIONALE**

Appropriate closure of the pancreatic remnant after distal pancreatectomy is still debated. A variety of procedures have been recommended to reduce the frequency of pancreatic fistula. Resections of the pancreas reaching the left side of the superior mesenteric vein are defined as distal pancreatectomy. They are performed less often than resections of the pancreatic head (20 to 30% of all pancreatic resections [[1](http://www3.interscience.wiley.com/cgi-bin/fulltext/110474340/#BIB1)]) due to a lower incidence of pancreatic disease in this part of the organ and later appearance of clinical symptoms in case of malignancy resulting in metastatic disease[[2](http://www3.interscience.wiley.com/cgi-bin/fulltext/110474340/#BIB2)]. However, continuously improving imaging and diagnostic techniques have resulted in an increase in the frequency of the procedure. Most resections are performed electively (84 per cent) for the following indications: chronic pancreatitis (24 per cent), other benign diseases (22 per cent), malignant diseases (18 per cent), neuroendocrine tumours (14 per cent) and cysts of the pancreas (6 per cent). The remaining 16 per cent are emergencies after abdominal trauma [[3](http://www3.interscience.wiley.com/cgi-bin/fulltext/110474340/#BIB3)]. In recent years the mortality rate after pancreatic resection has decreased considerably to between 0 and 6 per cent in high-volume centres[[1](http://www3.interscience.wiley.com/cgi-bin/fulltext/110474340/#BIB1)][[4](http://www3.interscience.wiley.com/cgi-bin/fulltext/110474340/#BIB4)][[5](http://www3.interscience.wiley.com/cgi-bin/fulltext/110474340/#BIB5)], but morbidity remains high, ranging from 10 to 47 per cent[[2](http://www3.interscience.wiley.com/cgi-bin/fulltext/110474340/#BIB2)][[5](http://www3.interscience.wiley.com/cgi-bin/fulltext/110474340/#BIB5)][[6](http://www3.interscience.wiley.com/cgi-bin/fulltext/110474340/#BIB6)]. Pancreatic fistula and leakage are the most common and clinically relevant complications, and they are thought to depend on surgical technique and skill [[2](http://www3.interscience.wiley.com/cgi-bin/fulltext/110474340/#BIB2)][[6-9](http://www3.interscience.wiley.com/cgi-bin/fulltext/110474340/#BIB6)]. Because fistula is associated with local and general complications (pancreatic fluid collection, formation of intra-abdominal abscesses, wound infection, delayed gastric emptying, respiratory complications, sepsis), it has important implications for the patient, the surgeon and the healthcare system. In general, it prolongs hospital stay for specialized treatment, including re-operation and drainage [[3](http://www3.interscience.wiley.com/cgi-bin/fulltext/110474340/#BIB3)][[10](http://www3.interscience.wiley.com/cgi-bin/fulltext/110474340/#BIB10)][[11](http://www3.interscience.wiley.com/cgi-bin/fulltext/110474340/#BIB11)]. The appropriate technique for closure of the pancreatic remnant is still debated. There is no evidence whether stapled or hand sewn closure should be preferred (DISPACT trial). Since stapled closure is not recommended for the triangular pancreas, the thick pancreas ( > 1.5 cm), the firm or non-compressible pancreas and in case of a resection plane to the right of the gastroduodenal artery, in this study we will use a standardized hand sewn closure in these patients.

Topical hemostatic agents applied to the resection surface of the remnant liver after partial liver resection have shown to be effective in preventing postoperative bleeding

[12]. The group of van Gulik showed that application of topical hemostatic agents, such as fibrinogen/thrombincoated collagen patch (TachoSil®, Nycomed, UK Ltd.) has a biliostatic effect, which can be considered an additional benefit of the use of these devices [13]. In addition, the adhesive strength of TachoSil on the resection surface, however, was superior to other agents. Results of retrospective studies on reconstruction techniques after pancreatico-duodenectomy suggest that outcome with regard to pancreatic fistula can be improved using TachoSil® [14].

## OBJECTIVES

The proposed study is a multicentre randomised controlled trial investigating the effect of Tachosil sealing of the pancreatic remnant after distal pancreatectomy on the development of pancreatic fistula.

Patients will be randomised to either Tachosil sealing of the pancreatic remnant after standardized hand sewn or stapled closure or conventional closure (hand sewn or stapled) alone.

Primary endpoint will be the development of pancreatic fistula.

Secondary endpoints will be overall mortality and morbidity and a cost effectiveness analysis.

###### STUDY DESIGN

Prospective randomized controlled multi-centre trial with blinding for treatment allocation of patients and medical personnel except for the surgeon. Patients will be evaluated according to the inclusion criteria at the outpatient clinic of participating centres. If eligible, informed consent is obtained. Inclusion will start after approval of Medical Research Ethical Committee.

## Conventional closure:

Hand sewn or stapled

Hand sewn is recommended for:	the triangular pancreas

the thick pancreas ( > 1.5 cm)

the firm or non-compressible pancreas

in case of a resection plane to the right of the gastroduodenal artery

## STUDY POPULATION

Patients who will undergo explorative laparotomy for planned distal pancreatectomy will be asked to enter the study. If a resection is carried out, based on preoperative findings, participants are eligible for randomization.

### ***Inclusion criteria***

- Age above 18 years
- Expected survival time more than 12 months
- WHO Karnofsky performance status >50%/ ASA I - II
- Patients with chronic pancreatitis, cystic pancreatic lesions, malignant pancreatic lesions and neuroendocrine tumours all located in the body or tail of the pancreas
- Patients who are planned to undergo distal pancreatectomy as part of an extensive resection for other malignacies (i.e. sarcoma, GIST, gastric carcinoma)
- Written informed consent

### ***Exclusion criteria***

- Current immunosuppressive therapy
- Chemotherapy within 2 weeks before operation
- Curative resection not feasible
- Severe psychiatric or neurologic disease
- Drug- and/or alcohol abuse

## SCREENING PROCEDURE FOR STUDY ENTRY

### The screening procedures consists of:

***History***

General medical history (previous disease, operations, allergies, other relevant factors), primary diagnosis (history, extent of disease), previous treatments for the primary diagnosis, secondary diseases and treatments for the secondary diseases

***Physical examination***

Total body examination, weight and Karnofsky performance status

***Special examinations***

- Tri-phasic spiral CT (pancreas, liver)
- Serum tumour marker Ca 19-9, CEA
- Usual laboratory values (blood count, plasmatic coagulation, total protein, electrolytes (Na, K, Ca, Cl), liver enzymes (total bilirubin, LDH, Alkaline phosphatase, GOT, GPT, GGT, renal functional parameters (serum creatinin, urea, uric acid, urine analysis creatinin), lipase/amylase
- MRI upper abdomen (facultative)
- Endoscopic ultrasound ± fine needle aspiration (facultative)
- ERCP (facultative)
- Laparoscopic staging (facultative)
- Abdominal ultrasound (facultative)

***Tumour measurement/assessment***

On spiral CT scan/MRI

###### SAMPLE SIZE

Sample size is calculated based on data from are own consecutive series of patients ( n=112) after distal pancreatectomy. Incidence of grade I to V pancreatic fistula was 30% in this cohort, which was also found in a recent meta-analysis [15]. By using Tachosil we expect to achieve an absolute reduction of pancreatic fistula of 15%. At α = 0.05 (two-sided) and β = 0.20, 118 patients are needed in each arm assuming 30% pancreatic fistula in the control group and a reduction of the fistula rate with 15% in the experimental group. Assuming a dropout of 10%, total sample size has to be 250 patients.

###### RANDOMISATION

An informed consent is signed prior to surgery. Randomisation is done intra-operatively after complete exploration of the abdomen and resection of the lesion in the pancreatic body or tail. Patients are randomised computer-based with stratification for centre. Patients and medical personal will be blinded for treatment allocation except for the surgeon performing the operation.

###### STANDARD TREATMENT PROTOCOLS

All patients will receive prophylactic antibiotics prior to surgery, together with a daily dose of subcutaneous low molecular weight heparine. Octreotide analogues are not administered as prophylaxis. Nasogastric suction tubes are placed during surgery. Peri- and post-operative pain management includes epidural anaesthesia or patient-controlled analgesia in all patients..

###### SURGERY

Abdominal approach can be achieved midline or by transverse laparotomy in line with local standards. A complete exploration of the peritoneal cavity, including frozen sections, is performed to refrain from resection in case of metastatic disease. Splenic preservation is attempted in case of benign or borderline tumours (i.e. cystic neoplasm). In case of malignancy a splenectomy should be performed.

Surgical procedures for both treatment arms are described below. No additional coverage of the pancreatic remnant is permitted.

***Conventional closure of the pancreatic remnant***

After complete mobilisation of the pancreatic body or tail (up to the region of the superior mesenteric vein, or at least 2-3 cm central of the planned resection margin), the resection is performed with a surgical scalpel or diathermia. Since unpublished data of the DISPACT trial did not show any significant difference between either hand sewing or stapler closure both procedures are allowed according to local practise. In case of hand sewing closure, after haemostasis the pancreatic duct is closed separately using PDS 4x0 or 5x0. The pancreatic remnant is closed using continuous mattress sutures with PDS 3x0 or 4x0.

***Closure of the pancreatic remnant by Tachosil seal***

In the Tachosil arm, pancreatic resection and transsection of the pancreatic body will be executed with a surgical scalpel or diathermia.and closure will be performed as described above , either hand sewing or with a stapler. The suture line is reinforced by placement of a fibrin/ thrombin-coated collagen patch (TachoSil) onto the transsected end . All participating centre receive a DVD where this procedure is demonstrated.

###### INTRA-OPERATIVE DRAINAGE

Each patient receives at least one intra-abdominal drainage tube in close proximity to the pancreatic remnant to be able to record amylase levels as well as drain output. The drainage tube used complies with local standards, but a soft, non-rigid drainage tube is recommended. On the second postoperative day serum amylase together with the amylase level in drain fluid will be measured. The tube should be removed no sooner than three days after surgery and removal must depend on the volume and content. Removal is recommended after 3 days when amylase levels are less than 3 times the serum level independent of it’s total production or a production of less than 50 mL/24 hr .

**OUTCOME MEASURES**

***Primary endpoint***

The primary outcome is the development of pancreatic fistula. A pancreatic fistula develops when the closure of the pancreatic remnant fails to heal, causing a leak of pancreas- derived, enzyme-rich fluid from the pancreatic ductal system.

A pancreatic fistula is defined as drainage of greater than 50 mL/24 hr amylase-rich fluid (> 3-fold elevation above upper limit of normal in serum) per day through the drains

on or after postoperative day 10.

Pancreatic fistulas will be graded according to international fistula definition as published by Strasberg based on the clinical impact on the patient’s hospital course.

***Grade 1*** Deviation from normal postoperative course without pharmacologic, endoscopic, surgical or radiologic intervention (certain drugs allowed)

***Grade 2*** Pharmacologic treatment needed. Includes blood transfusions and total parenteral nutrition

***Grade 3*** (a/b) Surgical, endoscopic or interventional radiologic treatment needed

a: Not under general anaesthesia	b: Under general anaesthesia

***Grade 4*** (a/b) Life threatening complications and organ dysfunction

a: Single organ	b: Multi-organ

***Grade 5*** Death due to pancreatic fistula

***Secondary endpoints***

Secondary endpoints are mortality, morbidity and costs.

The mortality rate is defined as the total in-hospital death rate.

Postoperative complications, especially delayed gastric emptying, which is defined as a nasogastric tube for more than 10 days after surgery, inability to proceed to a regular diet within 10 days or vomiting for more than 3 consecutive days after the fifth post-operative day.

Length of hospital stay

Direct in hospital medical costs (diagnostic procedures, surgical or radiological interventions)

***Additional study parameters***

Of all patients, baseline characteristics that might intervene with the main outcome parameter, such as gender, age, medical history, etc, will be documented. Special attention will be paid to operative variables, the extent and complexity of the procedure and final pathologic diagnosis.

###### FOLLOW-UP

Patients are followed up at day 10 post-operatively and at the day of discharge for both primary and secondary endpoints.

For evaluating secondary endpoints and survival, patients visit the outpatient clinic 30 days, 6 and 12 months after surgery.

Patient’s age, sex, indications for surgery, concomitant splenectomy, additional procedures, methods of pancreatic stump closure, and post-operative complications with a specific focus on pancreatic leak, mortality, and duration of post- operative hospital stay will be recorded.

Operative details such as operating time and the texture of the parenchyma are also recorded.

###### STATISTICAL CONSIDERATIONS

Analysis will be performed with the Statistical Package for the Social Sciences version 15.0 (SPSS). Mann-Whitney and Chi- square tests will be used for group comparisons and Student’s t-test for analysing normally distributed quantitative data.

Patients will be stratified for the texture of the parenchyma/ indication for surgery (chronic pancreatitis vs. the other lesions).

A p-value of < 0.05 is considered statistically significant.

## Descriptive statistics

All analyses will be based on the intention-to-treat principle. Baseline characteristics will be summarised using descriptive statistics.

## Univariate analysis

The principal analysis of this trial consists of an intention-to-treat comparison of postoperative incidence of pancreatic fistulas in both treatment strategies. The comparison will be expressed in terms of relative risk (RR) using 95% confidence intervals.

## Multivariate analysis

The primary outcome parameter will additionally be analyzed using multivariate logistic regression.

## Interim analysis

An interim analysis is planned when 50% of the patients are included (n = 125) to evaluate treatment effect. Because of the delayed response for determination of the primary outcome parameter, this analysis will be performed by the trial coordinator 30 days after randomization of the last patient to reach n = 125. The nominal significance (sequential two-sided testing) for the stopping rule is p <0.01 (alpha = 0.01).

# **ETHICAL CONSIDERATIONS**

## Regulation statement

This trial will be conducted according to the principles of the Declaration of Helsinki (version 2004) and in accordance with the Medical Research Involving Human Subjects Act (WMO) and other European guidelines, regulations and acts.

## Recruitment and consent

The patient will be asked to participate in the study at the outpatient clinic, when the indication for an explorative laparotomy is determined. Surgeons from the cooperating centres will inform the patients about the study and ask them for their consent. Patients will be given time to think about their decision until one week before the operation. They can reconsider their decision anytime.

The information offered to the patient or representative contains:

- a statement that the trial involves research
- a full explanation of the nature, expected duration, and purpose of the study
- a full and fair explanation of the procedures to be followed, identifying which of them are experimental or additional for study purposes
- a description of any reasonable foreseeable risks or discomfort to the patient
- a description of any benefits which may reasonably be expected
- a statement that participation is voluntary, that refusal to participate will involve no penalty or loss of benefits to which the patient is otherwise entitled, and that the patient may discontinue participation at any time without penalty or loss of benefits, in which case the patient will receive standard treatment with the same degree of care.

The patient information letter and informed consent form are attached as a separate document.

## Objection by minors or incapacitated subjects

Minors and incapacitated or legally incompetent adults are excluded from the trial.

## Benefits and risks assessment, group relatedness

No major risks or benefits are likely to be associated with participation in this study since Tachosil is already used very often by most of the surgeons without any known side-effect

## Incentives

Enrolled patients will not receive any special incentives, compensation or treatment through participation in this trial.

# ADMINISTRATIVE ASPECTS AND PUBLICATION

## Handling and storage of data and documents

For each randomised patient a case record form (CRF) will be completed. The CRF consists of a sequential set of instructions with provision for data recording.

The trial coordinator and local investigators will warrant that patients’ anonymity is maintained. Therefore, all randomised patients are identified by a Patient Identification Number (PIN) in combination with a center number. This number will be used on all screening forms, digital CRFs or other documents submitted to the coordinating center, instead of the patients’ name. The subject identification code list will be safeguarded by the trial coordinator. Trial personnel will not pass names outside the local hospital.

Central data management will be performed in paper and digital CRFs stored in a research file. All alterations on data entry will be explained using a red pen, noting the initials of the data-monitor as well as the date and reason for alteration. Old data will be crossed out in a way that it is still legible.

The trial coordinator will create a Trial Master File consistent with GCP-guidelines and will store this file in a secure but accessible manner. All essential documents will be legible and accurate. The participating centers will keep copies of relevant documents, including essential center-specific documents.

Randomization software will be made available by the department of Clinical Epidemiology and Biostatistics of the ErasmusMC.

When the trial is finished, all essential documents pertaining to the conduct of the trial (e.g., screening forms, CRFs, patient files, originals of test result reports, correspondence, records of informed consent, etc) will be archived by the trial coordinator for a period of 15 years in accordance with the standard operating procedure of the ErasmusMC.

## Amendments

Amendments are changes made to the research after a favourable opinion by the accredited METC has been given. All amendments will be notified to the METC that gave a favourable opinion.

All substantial amendments will be notified to the METC and to the competent authority.

Non-substantial amendments will be recorded and filed in the Trial Master File by the trial coordinator.

## Annual progress report

The trial coordinator will submit a summary of the progress of the trial to the accredited METC once a year. Information will be provided on the date of inclusion of the first subject, numbers of subjects included and numbers of subjects that have completed the trial, serious adverse events, other problems, and amendments.

## End of study report

The investigator will notify the accredited METC of the end of the study within a period of 8 weeks. The end of the study is defined as the moment that the last patient’s follow up is completed.
In case of premature termination of the trial, the trial coordinator will notify the accredited METC, including the reasons for the premature termination.
Within one year after the end of the study, the trial coordinator will submit a final study report with the results of the study, including any publications/abstracts of the study, to the accredited METC.

## Public disclosure and publication policy

The Steering Committee will be responsible for policy and decision-making and has the final responsibility for the scientific conduct of the study. It is composed of representatives of the investigators. The specific tasks of the Steering Committee are to improve study reports and papers for publication. The main paper will be published with the coordinating investigator as first author. All members of the CRP-trial study group will be listed as co-authors when more than 10% of all patients are included.

###### ETHICAL CONSIDERATIONS

The responsible investigator ensures that this study is conducted in agreement with the declaration of Helsinki and with the guidelines of good clinical practice issues by the European Union. Patient data will be used for the purposes of the study, and patient’s codes will be used instead of their full names. All patients will be informed about the nature of the study, and possible complications will be discussed. Furthermore, patients will be informed that participation is voluntary and that they are free to refuse further participation at any time without consequences for subsequent care. The patient will get sufficient time after obtaining this information to consider participation and then be asked informed consent, which will be written and documented in the patient file.

1. Balcom JH IV, Rattner DW, Warshaw AL, Chang Y, Fernandez-del Castillo C. Ten-year experience with 733 pancreatic resections: changing indications, older patients, and decreasing length of hospitalization. Arch Surg 2001; 136: 391-398.
2. Andren-Sandberg A, Wagner M, Tihanyi T, Lofgren P, Friess H. Technical aspects of left-sided pancreatic resection for cancer. Dig Surg 1999; 16: 305-312.
3. Lillemoe KD, Kaushal S, Cameron JL, Sohn TA, Pitt HA, Yeo CJ. Distal pancreatectomy: indications and outcomes in 235 patients. Ann Surg 1999; 229: 693-698.
4. Neoptolemos JP, Russell RC, Bramhall S, Theis B. Low mortality following resection for pancreatic and periampullary tumours in 1026 patients: UK survey of specialist pancreatic units. UK Pancreatic Cancer Group. Br J Surg 1997; 84: 1370-1376.
5. Buchler MW, Wagner M, Schmied BM, Uhl W, Friess H, Z'graggen K. Changes in morbidity after pancreatic resection: toward the end of completion pancreatectomy. Arch Surg 2003; 138: 1310-1314.
6. Fahy BN, Frey CF, Ho HS, Beckett L, Bold RJ. Morbidity, mortality, and technical factors of distal pancreatectomy. Am J Surg 2002; 183: 237-241.
7. Sheehan MK, Beck K, Creech S, Pickleman J, Aranha GV. Distal pancreatectomy: does the method of closure influence fistula formation? Am Surg 2002; 68: 264-267.
8. Bilimoria MM, Cormier JN, Mun Y, Lee JE, Evans DB, Pisters PW. Pancreatic leak after left pancreatectomy is reduced following main pancreatic duct ligation. Br J Surg 2003; 90: 190-196.
9. Adam U, Makowiec F, Riediger H, Trzeczak S, Benz S, Hopt UT. Distal pancreatic resection - indications, techniques and complications. Zentralbl Chir 2001; 126: 908-912.
10. Adam U, Makowiec F, Riediger H, Benz S, Liebe S, Hopt UT. Pancreatic leakage after pancreas resection. An analysis of 345 operated patients. Chirurg 2002; 73: 466-473.
11. Fernandez-del Castillo C, Rattner DW, Warshaw AL. Standards for pancreatic resection in the 1990s. Arch Surg 1995; 130: 295-299
12. Frilling A, Stavrou GA, Mischinger HJ, de Hemptinne B, Rokkjaer M, Klempnauer J, Thörne A, Gloor B, Beckebaum S, Ghaffar MF, Broelsch CE: Effectiveness of a new carrier-bound fibrin sealant versus argon beamer as haemostatic agent during liver resection: a randomised prospective trial. Langenbecks Arch Surg 2005; 390: 114–120.
13. D. Erdogan W. de Graaf T.M. van Gulik. Adhesive Strength of Fibrinogen-Coated Collagen Patch or Liquid Fibrin Sealant in an Experimental Liver Resection Model in Pigs. Eur Surg Res 2008;41:298–302.
14. Chirletti P, Caronna R, Fanello G, Schiratti M, Stagnitti F, Peparini N, Benedetti M, Martino G. Pancreaticojejunostomy with Applicationof Fibrinogen/Thrombin-Coated Collagen Patch (TachoSil(R)) in Roux-en-Y Reconstruction after Pancreaticoduodenectomy.J Gastrointest Surg. 2009 Apr 18.
15. Knaebel H.P., Diener MK, Wente MN, Büchler MW, Seiler CM. Systematic review and meta-analysis of technique for closure of the pancreatic remnant after distal pancreatectomy. Br J Surg 2005;92:539-546.
